# A cross‐sectional survey of moral distress and ethical climate – Situations in paediatric oncology care that involve children’s voices

**DOI:** 10.1002/nop2.1221

**Published:** 2022-04-20

**Authors:** Päivi Ventovaara, Margareta af Sandeberg, Gitte Petersen, Klas Blomgren, Pernilla Pergert

**Affiliations:** ^1^ 27106 Department of Women’s and Children’s Health Karolinska Institutet Stockholm Sweden; ^2^ Paediatric Haematology and Oncology Astrid Lindgren Children’s Hospital Karolinska University Hospital Stockholm Sweden; ^3^ Department of Paediatrics and Adolescent Medicine Copenhagen University Hospital (Rigshospitalet) Copenhagen Denmark

**Keywords:** ethical climate, moral distress, nurses, oncology, paediatric

## Abstract

**Aim:**

To assess experiences of morally distressing situations and perceptions of ethical climate in paediatric oncology care, with a focus on situations that involve children's voices.

**Design:**

Cross‐sectional survey.

**Methods:**

Registered Nurses at all four paediatric oncology centres in Denmark were asked to complete a web‐based questionnaire with Danish translations of the Swedish Moral Distress Scale‐Revised (MDS‐R) and the Swedish Hospital Ethical Climate Survey‐Shortened (HECS‐S). Data analysis included descriptive statistics and non‐parametric correlation tests.

**Results:**

Nurses (*n* = 65) perceived morally distressing situations as rather uncommon, except for those that involved shortage of time, poor continuity of care and unsafe staffing levels. Most nurses (83%) found it disturbing to perform procedures on school‐aged children against their will, and 20% reported that they do this often. Perceptions of ethical climate were positive and healthcare professionals were perceived to be attentive to children's wishes.

## 1 INTRODUCTION

Paediatric oncology is a rewarding speciality. However, it often exposes healthcare professionals to occupational stressors and distressing situations (Boyle & Bush, 2018) that challenge the provision of high‐quality, ethically good care. Some situations originate from organizational constraints, revealing themselves as understaffing (Rost et al., 2020), while others reflect difficulties in providing the best of care, such as not being able to relieve a child's pain (Pearson, 2013). Situations that prevent nurses from taking the action one considers as ethically right, can arouse negative feelings and anguish, also called moral distress (Boyle & Bush, 2018). Despite a proliferation of studies on moral distress, very few have been conducted in paediatric oncology.

## 2 BACKGROUND

The concept of moral distress was founded in 1984 although the ethical challenges had existed long before (Jameton, 2013). It arises from care situations when one does not carry out an action one considers as the ethically right thing to do (Boyle & Bush, 2018). Unattended moral distress can lead to burnout (Neumann et al., 2018), intention to leave (Colville et al., 2019) and to diminished quality of patient care (Henrich et al., 2017). Prior research has identified futile treatments and continuation of care not in the child's best interest as common causes of moral distress (Lazzarin et al., 2012). Many nurses also experience unsafe staffing levels and poor communication as morally distressing (Pergert et al., 2019). Institutional constrains that hinder healthcare professionals from acting in accordance with their moral values have aroused an interest to study the relationship between moral distress and ethical climate (Morley et al., 2019). Positive perceptions of ethical climate have been associated with less frequent experiences of moral distress (Silen et al., 2011; Ventovaara et al., 2021).

Ethical climate has been described as employees’ perceptions of ethically right ways of behaviour (Victor & Cullen, 1987), and as organizational practices on how ethical issues are discussed and decided (Olson, 1998). Prior studies have shown that ethical climate impacts the (un)ethical behaviour of employees (Treviño et al., 2014). When facing an ethical dilemma and having to decide about it, most adults seek external guidance from significant others, leaders, peers, rules and guidelines (Treviño et al., 2014). In paediatrics, the decision‐making should also be guided by an integrated child and family‐centred care, where the child's interests are primary and the parents contribute with their knowledge of their child and of the family's goals and values (Moynihan et al., 2021). Previous research has shown favourable impressions of ethical climates in healthcare (Koskenvuori et al., 2019), but paediatric healthcare professionals have also expressed difficulties in providing ethically good care (Bartholdson et al., 2016).

Many clinical situations in paediatric healthcare involve ethical dilemmas about what action benefits the child most (Bartholdson et al., 2015). Sometimes, the action that raises doubts about the child's best interest also involves overriding his/her wishes. Children have not yet achieved true autonomy, but according to the United Nations Convention on the Rights of the Child, they do have a right to participate in decisions about matters that affect them (Office of the United Nations High Commissioner for Human Rights [OHCHR], 1989). However, this right only applies to some extent, depending on the child's level of maturity and decision‐making capacity and thus, many healthcare decisions are made by children's proxy voices, parents and healthcare professionals. Children themselves are not always encouraged to participate in decision‐making processes (Quaye et al., 2019) and concerns about family‐centred care silencing children's voices have been raised (Davies et al., 2019). Even when children do express their wishes, they can be easily overpowered when in conflict with medical needs, for example when forced immobilization is used in young, non‐cooperative children during medical procedures. While physical restraint can be in the child's best interest in life‐threatening situations, the ethical justification becomes more blurred when applied in everyday practice.

Forced procedures cause ethical concerns for paediatric nurses, especially when the procedure increases the suffering of the child in situations with a little hope of cure or if the nurse disagrees with the necessity of the procedure (Bartholdson et al., 2015). Another ethical problem related to paediatric decision‐making occurs when young patients are not allowed to hear the truth about their illness (Bartholdson et al., 2015) or their imminent death (Hopia & Heino‐Tolonen, 2019). The patient advocacy is an essential part of the paediatric nurses’ role and situations that make it difficult to adhere to the wishes of the children, can be morally distressing. Considering the many ethical dilemmas and differing perspectives in paediatric oncology care, moral distress and ethical climate should be studied in further detail.

So far, most research on moral distress and ethical climate has been conducted in adult care settings, and in paediatric settings mainly among intensive care nurses (Karakachian & Colbert, 2019; Koskenvuori et al., 2019; Lamiani et al., 2017). Lack of research on moral distress and ethical climate in paediatric oncology care led to a research project investigating these topics in the Nordic countries of Sweden, Finland, Denmark, Norway and Iceland (af Sandeberg et al., 2020; Pergert et al., 2019; Pergert, Bartholdson, Blomgren, et al., 2019; Ventovaara et al., 2021). As a part of this project, this study was conducted in paediatric oncology centres in Denmark. The aim was to assess nurses’ experiences of morally distressing situations and to investigate perceptions of ethical climate. The focus was on situations related to paediatric decision‐making that involve children's voices. The study instruments included three child‐specific items of particular interest. These assess nurses’ experiences of limited truth‐telling despite an active request from a child, performing procedures against children's will and nurses’ perceptions of how often children's wishes are taken into account. As the prior studies from Sweden and Finland have indicated that nurses often struggle with busy work‐shifts (Pergert, Bartholdson, & af Sandeberg, 2019; Ventovaara et al., 2021), an additional interest was to examine if shortage of time and unsafe staffing levels are related to the situations that involve children's voices.

### 2.1 Research questions

1. How do the nurses in paediatric oncology perceive the levels of frequency and disturbance of the different situations that can generate moral distress?
2. Are the frequencies of the situations that involve shortage of time and unsafe staffing levels related to how often nurses perform procedures against school‐aged children's will and how often nurses withhold from talking about death with children who ask about it?
3. How do the nurses in paediatric oncology perceive the ethical climate?
4. Are the wishes of children with cancer commonly taken into account in paediatric oncology care, according to the nurses’ perceptions?
5. Are the perceptions of morally distressing situations correlated with the perceptions of the ethical climate?

## 3 THE STUDY

### 3.1 Design

This is a cross‐sectional, Nordic Society for Paediatric Haematology and Oncology (NOPHO) and Nordic Society of Paediatric Oncology Nurses (NOBOS), study.

### 3.2 Method

#### 3.2.1 The questionnaire

A questionnaire, including socio‐demographic items, the Swedish Moral Distress Scale‐Revised (MDS‐R) (af Sandeberg et al., 2017), and the Swedish Hospital Ethical Climate Survey‐shortened (HECS‐S) (Pergert et al., 2018) was translated into Danish and applied in the present study. The original versions of these instruments were developed in the 1990s, the MDS by Corley et al. (2001) and the HECS by Olson (1998). Numerous healthcare researchers have used these instruments, or versions based on the originals (Giannetta et al., 2020; Koskenvuori et al., 2019).

The Swedish version of the questionnaire was translated into Danish as described below. Two translations were performed, one by a certified translator with no knowledge of paediatric oncology and the other one by a physician and a nurse (the third author) in paediatric haematology/oncology with Danish as their native language. An expert review board, consisting of healthcare professionals (*n* = 6) with extensive knowledge in paediatric oncology care, synthesized the two versions in relation to the Swedish questionnaire and to the English back‐translated version of it, into one. The discussions were led by a moderator and an observer was taking field‐notes. The synthesized version was used in think‐aloud interviews (*n* = 3) performed by the third author with two nurses and one physician. The aim of the think‐aloud interviews was to assess participants’ understanding and interpretation of items. The analysis of the interviews led to minor adjustments of the questionnaire. The Swedish questionnaire targets nurses, physicians and nursing assistants. However, in Danish paediatric oncology, there were no nursing assistants working in direct patient care and thus the Danish questionnaire does not include any items directed to nursing assistants.

*The Danish Moral Distress Scale – revised*consists of 26 items that describe situations that healthcare professionals can experience as morally distressing. Two of the described situations directly conflict with children's wishes, namely, *‘follow family's request not to talk about death with a dying child who asks about dying’*, and *‘perform painful/unpleasant procedures on school‐aged children who resist such treatment’*. Each item is assessed twice: firstly, by its level of disturbance (‘how would this situation affect you?’) and secondly, by its frequency (‘how often have you experienced this situation?’). Both assessments are scored on a scale from 0 (not at all/never) to 4 (very negatively/very often). Higher scores imply higher levels of moral distress.

*The Danish Hospital Ethical Climate Survey – shortened*consists of 18 items that describe conditions for ethical practices and relations between co‐workers, the hospital, the management and patients. This study focused on the item describing relations to patients, which reads *‘patient's wishes are taken into account’*. All items are scored on a scale from 1 (almost never) to 5 (almost always). Higher scores imply more positive perceptions of the ethical climate.

#### 3.2.2 The psychometric properties of the questionnaire

The Swedish versions of the MDS‐R and the HECS‐S were validated in the preceding studies in Sweden, providing evidence on content validity of the instruments (af Sandeberg et al., 2020; Pergert, Bartholdson, & af Sandeberg, 2019; Pergert, Bartholdson, Blomgren, et al., 2019). A Cronbach's alpha coefficient of 0.88 for the Danish HECS‐S (18 items), and 0.86 for both the frequency and the disturbance scales of the Danish MDS‐R (26 items) indicated good internal consistency.

#### 3.2.3 Data collection

The data collection was conducted during December 2019 and March 2020. All 137 nurses and 28 physicians who were working in direct patient care at the four paediatric oncology centres in Denmark during the data collection period were invited to participate. This study presents exclusively nurses’ responses and the results from the physicians will be reported elsewhere. Data were collected by using a web‐based questionnaire in the Artologik Survey & Report, Version 4.3 (Artisan Global Media). Two of the centres chose to use a public web link to the questionnaire. A contact person at these centres received the link to the questionnaire and forwarded it, together with an invitation to participate, to the potential participants who then could access the survey by clicking on the link. The public link did not allow the participants to save their answers and to continue the survey at a later time. Two other centres chose personal links to the questionnaire, meaning that all links were individual and unique for each potential participant. The use of private links allowed the participants to save their answers and to complete the questionnaire at a later time. Three reminders were sent in order to enhance the response rates.

### 3.3 Analysis

The IBM SPSS Statistics 25 was used to analyse the data. The instruments with more than 10 percent missing answers were excluded from the analysis. To describe central tendencies, a participant mean value was calculated for each scale. The Spearman's Correlation was used to examine the strength of association between items and between scales. Counts and percentages were calculated to examine distributions of the item scores. The 5‐point instrument scales were then dichotomized into mutually exclusive categories. In the present analysis, the middle option was regarded as neutral and the two options above it were considered to present positive perceptions of ethical climate (scores >3) and frequent/disturbing experiences of moral distress (scores >2). The major benefit of dichotomization is that it very much simplifies the presentation of the results (Farrington & Loeber, 2000). A Cronbach's alpha coefficient above 0.70 was taken as indication of an acceptable internal consistency (Mackridge & Rowe, 2018). In correlation analysis, a *p *< .05 was regarded as statistically significant.

### 3.4 Ethics

The instruments were used with permission from the developers (AB Hamric, Dr, email conversation, October 6, 2014; L Olson, Dr, email conversation, November 15, 2014, and May 29, 2015). According to the Act on Research Ethics Review of Health Research Projects (Danish Ministry of Health & the Elderly, 2018), the study was not of such a nature that notification was needed to the research ethics committee system (ML Nielsen, The Danish Council on Ethics and National Committee on Health Research Ethics, email conversation, February 1, 2018). The study followed the ethical principles of Declaration of Helsinki for conducting research involving human subjects (World Medical Association, 2018).

At a national paediatric oncology meeting, the third author provided oral information about the study to the nurses, the physicians and the managers of the four paediatric oncology centres. After the meeting, an invitation letter including information about the study was sent by email to the managers at the four centres, and all consented. The first page of the survey included information about confidentiality, that it was voluntary to participate, and the General Data Protection Regulations. The participants were asked to confirm that they had read and agreed with the terms and conditions, and only participants who did so, could answer the questionnaire.

## 4 RESULTS

Altogether, 66 nurses out of 137 (48%) completed the questionnaire. One participant was excluded from the Moral Distress analysis because more than 10% of the items were left unanswered. Table 1 provides an overview of the demographic characteristics of the participants.

**TABLE 1:** Demographic characteristics of the participants, *n* = 66

	*n*(%)
Gender	
Female	65 (98)
Male	—
Missing	1 (2)
Continued education	
Yes	22 (33)
No	43 (65)
Missing	1 (2)
Work experience in paediatrics	
<5 years	31 (47)
≥5 years	35 (53)

### 4.1 Moral distress

The situations described in the Danish MDS‐R were found to be highly disturbing (mean 3.21; *SD* 0.46) but most situations were reported to occur quite rarely, the mean frequency of all 26 items being 1.23 (*SD* 0.49) on a scale of 0 to 4.

As seen in Table 2, the three situations that were reported to occur frequently by the highest number of nurses, reflected shortage of time, impaired care quality due to poor continuity of care and unsafe staffing levels.

**TABLE 2:** The Danish MDS‐R items and the proportion of nurses who selected an option above the middle score (>2), suggesting frequent/disturbing experiences of moral distress. The two child‐specific items are marked with †. *N* = 65

Abbreviated items	Scores >2 *n*(%)	
	Frequency *n*(%)	Disturbance *n*(%)
Not having time to conduct conversations with patients and families in a way you think they should be carried out	28 (43)	55 (85)
See that … care suffers because of lack of continuity, with many different healthcare providers	25 (38)	52 (80)
Work in a staffing situation (number/competence level) that you experience as unsafe	22 (34)	58 (89)
Work with nurses … not as competent as … healthcare requires	15 (23)	55 (85)
Be unable to provide best possible care … pressures from management to reduce costs	15 (23)	57 (88)
^†^Perform painful/unpleasant procedures on school‐aged children who resist such treatment	14 (22)	54 (83)
Follow family's wishes… life‐sustaining treatment…not in the best interest of the child	11 (17)	46 (71)
Be expected to care for patients… not feel competent enough to care for	9 (14)	56 (86)
Feel pressured to perform tests and treatments … unnecessary	9 (14)	37 (57)
Provide care although parents have unrealistic expectations of healthcare	7 (11)	32 (49)
See that the quality of patient care suffers because of poor communication within the team	7 (11)	57 (88)
See inexperienced…professionals perform painful procedures… solely to improve skills	7 (11)	53 (82)
^†^Follow family's request not to talk about death… dying child who asks about dying	6 (9)	62 (95)
Continue to participate in life‐sustaining treatment of a dying child because no one has decided to end that treatment	6 (9)	53 (82)
Provide inadequate care…not relieve…suffering…physician afraid…will lead to death	6 (9)	57 (88)
Work with a physician… incompetent in providing healthcare	5 (8)	61 (94)
See… professionals give ‘false hope’ to parents	5 (8)	55 (85)
Avoid reporting… discover that a physician or a nurse has made a medical error	5 (8)	45 (69)
Not to talk about death with a dying child although you think it is necessary	4 (6)	56 (86)
Take life‐saving actions… only prolong dying	4 (6)	50 (77)
Decide on care/treatment when you are uncertain about what is right	3 (5)	45 (69)
Shut eyes to that parents have not received… information… to give their consent to health care…	3 (5)	51 (78)
Take no action about an ethical issue…because the involved…professional or management requests…	3 (5)	55 (85)
Follow the family's wishes for the child's care despite…you disagree…afraid of being reported	2 (3)	52 (80)
Follow physician's request not to discuss… prognosis with parents	2 (3)	49 (75)
Give an increased dose of sedatives/opiates… believe… hasten death	1 (2)	27 (42)

*‘Perform painful/unpleasant procedures on school‐aged children who resist such treatment’*was perceived to be very disturbing by most nurses. Every fifth nurse frequently experienced this situation at work (Table 2).

Most nurses found the situation *‘follow family's request not to talk about death with a dying child who asks about dying’* very disturbing. This situation ranked as the most disturbing one out of the 26 situations and six nurses reported they frequently experienced it (Table 2).

### 4.2 Ethical climate

Rated on a scale of 1 to 5, the ethical climate scale displayed a mean of 3.93 (*SD* 0.58). The majority of the nurses had selected a positive option (>3) in 15 out of 18 statements (Table 3). According to the nurses’ perceptions parents’ wishes were taken into account more often than children's wishes.

**TABLE 3:** The Danish HECS‐S items and the proportion of nurses who had selected an option above the middle score (>3), suggesting positive perceptions of ethical climate. The child‐specific item is marked with †. *N* = 66

	Scores >3 *n*(%)
Abbreviated items	
Co‐workers listen	63 (95)
Competent co‐workers	60 (91)
Parents’ wishes	60 (91)
Physicians and nurses trust	58 (88)
^†^Patients’ wishes	51 (77)
Openness asking questions, learning	51 (77)
Manager I trust	51 (77)
Physicians and nurses respect each other's opinions	51 (77)
Hospital's values shared	49 (74)
I can practise care as it should be	44 (67)
Feelings and values taken into account	42 (64)
Immediate manager helps	38 (58)
Physicians ask nurses for their opinions	38 (58)
Ethical problems identified	38 (58)
Immediate manager helps my co‐workers	34 (52)
Dealing with ethical problems	33 (50)
Hospital policies help	29 (44)
Conflicts openly dealt with	27 (41)

### 4.3 Correlation between items

A weak, positive correlation was found between the reported frequencies of *‘not having time to conduct conversations with patients and families in a way you think they should be carried out’* and two of the child‐related items *‘follow family's request not to talk about death with a dying child who asks about dying’* (*r *= 0.263, *n* = 62, *p* =.039) and *‘perform painful/unpleasant procedures on school‐aged children who resist such treatment’* (*r* = 0.385, *n* = 63, *p *= .002). Frequencies of inadequate staffing did not correlate with situations involving children's voices.

### 4.4 Correlation between moral distress and ethical climate

Perceptions of ethical climate were inversely correlated with the frequencies of morally distressing situations (*r* = −0.523, *n* = 65, *p* < .001). No correlation was found between the ethical climate and the disturbance levels of morally distressing situations.

## 5 DISCUSSION

The purpose of this study was to explore nurses’ experiences of moral distress and their perceptions of ethical climate in paediatric oncology care in Denmark, with the focus on situations that involve children's voices. Nurses found morally distressing situations as very disturbing but mostly quite uncommon. Only few nurses often withhold talking about death with a dying child, while every fifth nurse often performed procedures on school‐aged children without their approval. The findings also showed positive perceptions of ethical climates and a great attentiveness to children's wishes.

Almost all nurses perceived it as highly disturbing to give in to the family's demand not to discuss death with a dying child who asks about it. Although parents and nurses may share a desire to protect the child by concealing the harsh truth or by using white lies (Shali et al., 2020), the distressing impact of limited truth‐telling is well established in qualitative studies (Hopia & Heino‐Tolonen, 2019; Testoni et al., 2020). Consistent with earlier results (Ventovaara et al., 2021), nurses in this study did not experience this situation very often. Due to the way the situation is described in the MDS‐R, it cannot be concluded whether parents only rarely asked nurses to keep silent about death or if the nurses chose not to obey such requests. The astonishing survival rates in high‐income countries (Erdmann et al., 2021), however, support the idea of limited exposure to the described situation in general.

Procedures against children's will is a common ethical challenge in paediatric healthcare (Lombart et al., 2020) as not all children understand the importance of medical procedures and treatments. While it usually is children under 4 years who are restrained during procedures (Kirwan & Coyne, 2017), the present results show that every fifth nurse often carries out procedures without school‐aged children's cooperation. This accords with earlier findings from those studies that have included this child‐specific item in the Moral Distress Scale and have shown that performing procedures is one of the most common distressing situations in paediatric oncology nursing (af Sandeberg et al., 2020). Nurses have described forced procedures as an infringement on children's autonomy (Bartholdson et al., 2015), and therefore it can be assumed that it could be more morally distressing to use restraint on school‐aged children than when applying it to toddlers. Considering the number of procedures that children with cancer must undergo, it is important to gain children's cooperation and to help them to cope with distressing situations. The study by Kleye et al. (2021) emphasizes the importance of listening to children's voices as they already may have their own strategies for mastering procedures. Further studies among school‐aged children could shed light on their experiences in more detail and help nurses to better prepare children for procedures, which in turn could diminish moral distress associated with these situations.

In line with previous findings (Pergert, Bartholdson, & af Sandeberg, 2019; Sauerland et al., 2015), the most common morally distressing situations appeared to be related to shortage of time, lack of continuity and unsafe staffing levels. None of these directly involves children's voices. The Danish MDS‐R includes only two items that describe morally distressing situations where children actively voice their views. However, it is important to bear in mind that children's views and thoughts are constantly present in healthcare even when not expressed aloud.

Almost half of the nurses often experienced trouble finding sufficient time for discussions with patients and their families. The analysis revealed an association between shortage of time for having conversations and how often nurses performed procedures against school‐aged children's will and how often they did not reply the child's question about death. It is possible to hypothesize that shortage of time even contributes to limited communication with school‐aged children prior to procedures. This assumption is supported by Bray et al. (2019) who found that the need for holding children against their will can be reduced by devoting time to inform and to prepare children for procedures. Typically, time scarcity reflects high workloads and poor staffing levels. Surprisingly, unsafe staffing levels were not associated with the two morally distressing situations that involve children's voices. Although the lack of correlation was unexpected, it could be due to the definition of unsafe staffing levels. In the Danish MDS‐R, ‘unsafe staffing situation’ refers both to the number of staff members and to their competence.

The majority of nurses perceived the ethical climate at their workplaces as positive. Positive perceptions of ethical climate are very much in line with previous studies (Koskenvuori et al., 2019). According to nurses’ perceptions, parents’ wishes were slightly more often taken into consideration than children's wishes. Considering that many children diagnosed with cancer in Denmark are under 4 years or age (Grabas et al., 2020) and those parents have the authority to act as proxy decision makers, this finding is not unexpected. This outcome is in line with Swedish studies (Bartholdson et al., 2016; Pergert, Bartholdson, Blomgren, et al., 2019) but it differs from the results from a Finnish study that suggested equal consideration to parents’ and children's wishes (Ventovaara et al., 2021). This inconsistency may partly be due to instrumental errors in surveying, as these two response options are not mutually exclusive in a family‐centred model of care where children's and parents’ wishes often are intertwined. It is important to bear in mind that listening to children's voices does not necessarily mean granting children autonomy in decision‐making, but rather recognizing their integrity and allowing them to express their views and feelings.

In accordance with previous studies (Silen et al., 2011; Ventovaara et al., 2021), the present findings link perceptions of positive ethical climate with less frequent experiences of morally distressing situations. Although cross‐sectional studies cannot explain the causality, it can be assumed that while it is important to mitigate the experiences of moral distress, efforts should also be made to enhance the communication, the ways of dealing with ethical issues and other aspects of ethical climate.

### 5.1 Clinical implications

The combination of our current findings and previous empirical research calls for an ethical climate that inspires nurses to actively listen to children. Acknowledging children's voices could prevent some of the morally distressing situations, but as it can be quite time‐consuming, it can be difficult to adhere to if nurses already struggle with time constraints. The challenge now is to improve nursing workloads and staffing issues. Silen et al. (2012) suggest managers to give their support by staffing up when there are heavy workloads. In paediatric nursing this might involve taking one step further, as optimal staffing levels are not only about the nurse–patient ratios. Considering the long cancer treatments and frequent hospitalizations, a continuity of care is warranted. Higher baseline staffing could reduce moral distress, and even grant time for a child‐friendly approach, assuring that children's voices would be heard even in peak hours.

## 6 LIMITATIONS

Although this was a multi‐centre study, the number of potential study participants was limited to start with, and the low response rate makes the generalizability uncertain, so all results must be interpreted with caution. Furthermore, the modest sample size discouraged from some statistical tests such as group comparisons.

The ethical climate is a characteristic of a workplace, and it might have been more accurate to compare the perceptions within and between centres. However, due to the small number of participants in two of the centres this would have jeopardized confidentiality.

## 7 CONCLUSION

The results of this study showed that shortage of time for having conversations is a common cause of moral distress among paediatric oncology nurses. Paediatric nurses are attentive, even if they are slightly more likely to listen to parents’ than children's views. Although parents’ opinions weight more in medical decisions, children's views should not be overlooked. Ensuring resilient baseline staffing levels and maintaining continuity of care could prevent morally distressing situations and nourish an ethical climate where children are encouraged to voice their views.

## CONFLICT OF INTEREST

The authors declare no conflict of interest.

## AUTHOR CONTRIBUTIONS

**Päivi Ventovaara**: Formal analysis; Writing – original draft, editing. **Margareta af Sandeberg**: Conceptualization; Data curation; Funding acquisition; Project administration; Supervision; Validation; Writing – review and editing. **Gitte Petersen:** Data curation; Writing – review and editing. **Klas Blomgren:** Funding acquisition; Supervision; Writing – review and editing. **Pernilla Pergert**: Conceptualization; Data curation; Funding acquisition; Project administration; Resources; Supervision; Validation; Writing – review and editing.

## Data Availability

Data available on request from the authors.
